# The Bone Marrow Microenvironment as Niche Retreats for Hematopoietic and Leukemic Stem Cells

**DOI:** 10.1155/2013/953982

**Published:** 2013-01-10

**Authors:** Felix Nwajei, Marina Konopleva

**Affiliations:** ^1^Department of Immunology, The University of Texas MD Anderson Cancer Center, 1515 Holcombe Boulevard, Unit 0902, Houston, TX 77030, USA; ^2^Graduate School of Biomedical Sciences, The University of Texas Health Science Center at Houston, 6767 Bertner Avenue, 3rd Floor, Houston, TX 77030, USA; ^3^Department of Leukemia, The University of Texas MD Anderson Cancer Center, 1515 Holcombe Boulevard, Unit 428, Houston, TX 77030, USA

## Abstract

Leukemia poses a serious challenge to current therapeutic strategies. This has been attributed to leukemia stem cells (LSCs), which occupy endosteal and sinusoidal niches in the bone marrow similar to those of hematopoietic stem cells (HSCs). The signals from these niches provide a viable setting for the maintenance, survival, and fate specifications of these stem cells. Advancements in genetic engineering and microscopy have enabled us to critically deconstruct and analyze the anatomic and functional characteristics of these niches to reveal a wealth of new knowledge in HSC biology, which is quite ahead of LSC biology. In this paper, we examine the present understanding of the regulatory mechanisms governing HSC niches, with the goals of providing a framework for understanding the mechanisms of LSC regulation and suggesting future strategies for their elimination.

## 1. Introduction

A dysfunctional stem cell microenvironment, or niche, contributes significantly to disease pathology, particularly in cancer [1]. Characterization of the cells that form this niche and the mechanisms by which they regulate stem cell function is imperative for understanding the pathophysiology of diseases that arise in this setting. Stem cells have the unique ability to self-renew, differentiate into multiple lineages, and withstand stress signals to survive and function [2, 3]. In the bone marrow, hematopoietic stem cells (HSCs) are essential for the production of both lymphoid and myeloid cells, which are necessary for the body's immune integrity, oxygen delivery, blood clotting, waste removal, and a multitude of physiologic processes necessary for survival.

For some time, the intracellular regulatory environment of HSCs has been studied in the isolation of its confines in the bone marrow, with little emphasis on the effects this environment might have on these cells' survival and fate specifications [4]. Testing of the prevailing theory, proposed by Schofield, regarding the underlying indispensable role of the bone marrow structure in engineering hematopoiesis [5] became possible only with the advent and introduction of new *in vivo* technological tools such as intravital multiphoton microscopy (IVM), which is powerful in optical sectioning of deep tissues and providing real-time visualization of cellular interactions [5, 6]. This has led to a radical revolution in the way stem cells are studied in the bone marrow. IVM studies have shown that hematopoiesis depends not only on the cellular biology of HSCs, but also on the microenvironment where they reside, buttressing the “stem cell niche” hypothesis [5].

HSCs reside in two distinct niches in the bone marrow, the endosteal and vascular [7–13]. These niches are complex, encompassing a broad range of bone marrow cells that includes bone lining cells (osteoblasts and osteoclasts), mesenchymal stem cells (MSCs), sinusoidal endothelium and perivascular stromal cells, immune cells, and several others that play different roles in HSC regulation [14]. In the context of the “seed and soil” hypothesis, studies in solid organs of nonhuman mammals have shown that MSCs fuel the growth of cancerous cells and contribute to the therapy resistance and metastatic potential of tumors by shielding cancer stem cells [15–18]. However, because of its anatomy, the bone marrow is a more complex system that includes both an endosteal bone surface stem cell microenvironment and a vascular niche.

**Figure 1:**
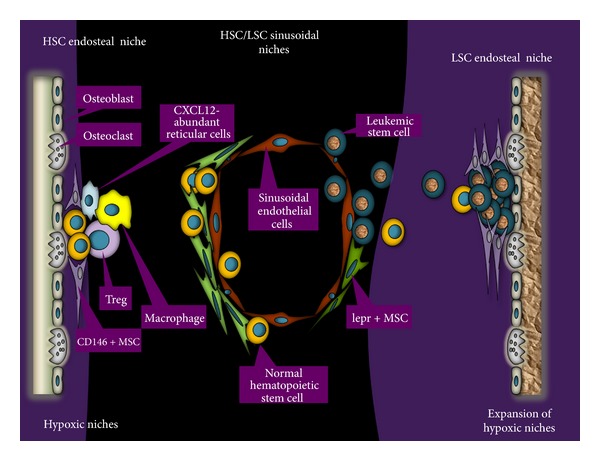
Organization of normal hematopoietic stem cell (HSC) and leukemic stem cell (LSC) niches in the bone marrow. Both HSCs and LSCs establish niches around the bone marrow endosteum and sinusoids. In normal hematopoiesis, the endosteal niche is formed and regulated by osteoblasts, osteoclasts, mesenchymal stromal cells (MSCs), T-regulatory cells (Tregs), and macrophages, while in leukemia, LSCs associate with osteoblasts and mesenchymal stromal cells. HSCs form sinusoidal niches with sinusoidal endothelial cells and leptin receptor-(lepr+-) expressing-perivascular stromal cells. LSCs form sinusoidal niches with sinusoidal endothelial cells. Oxygen gradient decreases from the sinusoids to the endosteum. The normal HSC endosteal niches are hypoxic, while there is an expansion of hypoxic niches in LSC endosteal niches due to LSC proliferation.

The biology of HSCs shares many similarities with that of leukemia stem cells (LSCs). Despite these similarities, LSCs are able to outcompete HSCs, hijacking the bone marrow microenvironment and subverting it to a relatively more hypoxic state suitable for their survival and proliferation [19–22] (illustrated in Figure 1). Previous review articles from our group and others have critically analyzed the role of the bone marrow microenvironment in acute myeloid leukemia (AML) [23–25]. In this paper, we dissect the biology of HSC niches and the impact of the immune system, oxygenation/hypoxia, and MSCs on the maintenance of HSCs. In this context, we discuss LSC niches, using chronic myeloid leukemia (CML) as a model and providing insight into potential therapeutic strategies.

## 2. Niche Retreats for HSCs in the Bone Marrow

The bone marrow endosteum and sinusoids are the two predominant niches for HSCs. Prevailing early studies had suggested the bone marrow endosteum as the major HSC niche. This was demonstrated by utilizing hematopoietic progenitor cells stained with nonspecific markers that did not specifically label HSCs. However, *in vivo* studies of HSCs became possible when it was discovered that a unique array of surface adhesion markers, the signaling lymphocyte activation molecule (SLAM) family receptors, comprising CD150^+^CD244^−^CD48^−^, could be used to select and purify HSCs with great specificity [10]. With this capability, most HSCs were shown to reside adjacent to sinusoidal endothelium in the spleen and bone marrow, while a few were observed to show preference for the bone marrow endosteum, as shown in Figure 1. Therefore, two specialized niches in the bone marrow support HSCs. Elucidating the functions of these two niches is crucial to the understanding of the behavior of HSCs and to exploiting this knowledge for clinical applications.

### 2.1. Endosteal HSC Niche

The endosteum is the inner surface of the bone marrow cavity, made up of both cortical and trabecular bone types, where hematopoiesis occurs actively. This surface is lined by bone cells such as osteoblasts and osteoclasts. Osteoblasts are progenitor bone-forming cells that work in tandem with osteoclasts in the process of osteogenesis [26]. They are transient cells that actively provide mineralization during bone development and replace lost bone tissues in adults. IVM revealed the homing of fluorescently labeled HSCs to the bone marrow endosteum, suggesting a preference for this anatomical site for their survival and maintenance [14]. Previous studies have shown direct associations between osteoblasts and HSCs. For instance, in a fascinating study conducted by Chan and colleagues, the knockdown of osterix, an osteoblast-specific transcription factor, essential for endochondral ossification, led to impairment of bone formation and the absence of the HSC niche at an ectopic kidney site [27]. Again, HSC number was decreased by conditionally depleting osteoblasts in transgenic mice [28]. However, an expansion in the osteoblast number requires other factors to mediate a proportionate increase in the HSC pool [29]. Some of these factors can be expressed by osteoblasts *in vitro*, including several cytokines, chemokines, and adhesion molecules, such as CXCL12, angiopoietin-1, stem cell factor (SCF), and thrombopoietin, to maintain HSCs. In the process of validating some of these *in vivo*, in conditional knockout mice, Ding and colleagues demonstrated that conditional deletion of SCF from osteoblasts does not affect HSC number, whereas its deletion from endothelial and leptin receptor- (lepr-) expressing perivascular stromal cells significantly reduces HSC number [9]. This suggests that the regulation of HSCs in their niche is very cell specific, or rather niche specific. The HSCs that associate with osteoblasts are quiescent in nature, giving them the ability to survive and contribute to hematopoiesis over a long period of time [30–33].

Osteoclasts are bone-resorbing cells that coordinate with osteoblasts in bone formation [26]. They are less well characterized than osteoblasts in the context of HSC niche formation and maintenance. Nevertheless, new findings are beginning to emerge on the role of osteoclasts in the process of hematopoiesis. Osteoblast expansion had been observed to cause a proportionate increase in HSC number [8, 13]. Lymperi and colleagues demonstrated, however, that HSC number did not increase on administration of strontium, an element with the dual effects of osteoblast expansion and osteoclast depletion [34]. They hypothesized that this observation could be explained by the reduction in osteoclast number and activity. In line with this, they showed that bisphosphonates inhibit osteoclasts in mice and that this inhibition severely depresses HSCs and delays hematopoietic recovery [35]. More recently, osteoclast impairment reduced osteoblast differentiation and HSC localization in the oc/oc mouse model, in which endochondral ossification is impaired because of osteoclast deficiency [28]. These findings reveal a greater complexity of hematopoiesis regulation than was previously known; more studies are needed to clarify how osteoclastic involvement in this process connects to the better understood osteoblast involvement.

### 2.2. Immune Privilege

Regardless of the bone marrow's role in the production of immune cells, which maintain the immune integrity of the body, there is limited data on the activity of immune cells in the HSC microenvironment in the bone marrow. An IVM study suggested that regulatory T cells (Tregs) contribute to the formation of a localized zone and relative sanctuary for hematopoietic stem/progenitor cells (HSPCs), which provides a safe environment for HSPC maintenance and survival from immune attacks [14]. In that study, HSPCs from allogeneic donor mice survived as long as 30 days in nonirradiated immunocompetent mice, similar to the survival of syngeneic HSPCs. IVM revealed that Tregs lodge around the HSPC microenvironment in the bone marrow endosteum and protect them by creating an immune privilege sanctuary akin to those in the testis, eye, and brain as depicted in Figure 1. These HSPCs were lost following depletion of Tregs [14]. In a different study, depletion of macrophages disengaged HSCs from their endosteal niche into the circulation by reducing osteoblast number and cytokines that mediate the adhesion of HSCs to this niche [36, 37]. Our increasing understanding of the role of immune activity in the HSC niche shows great promise for development of novel strategies that will be more effective than the current approaches in harvesting HSCs and in preventing graft-versus-host disease in patients undergoing HSC transplant for a hematologic malignancy.

### 2.3. Sinusoidal HSC Niche

Bone marrow sinusoids are thin-walled vessels that serve as the medium for communication between the marrow cavity and blood circulation. They are lined by a single layer of endothelium and directly continue from arterioles to venules. A broad range of cells, including adventitial reticular cells, perivascular stromal cells, MSCs, and neurons, associate with sinusoids to form a niche that can sustain and regulate HSCs. Identification of HSCs in this niche (by the SLAM family receptors CD150^+^CD244^−^CD48^−^ [10]) provided a link between the maintenance of HSCs in sinusoidal niches of the liver/spleen and the bone marrow and suggested that the HSC niche is perivascular. The cells that support this niche have been suggested to express a range of cytokines, such as SCF, CXCL12, and alkaline phosphatase, which have been shown to support the maintenance of HSCs *in vitro*.

Uncertainty about which specific sinusoidal or endosteal niche cell is functionally important in producing any of these molecules and sufficient to maintain HSCs led Ding et al. to conduct the study already mentioned in which cre-lox conditional knockout mice were used to delete SCF *in vivo* from osteoblasts, sinusoidal endothelium, perivascular stromal cells, and nestin-positive MSCs [9]. Their results showed that sinusoidal endothelium and lepr-expressing perivascular stromal cells, but not osteoblasts or nestin-cre-or nestin-creER-expressing cells, are directly responsible for the expression of SCF and functionally regulate HSCs in the sinusoidal niche; their deletion resulted in decreased hematopoiesis in the liver, spleen, and bone marrow. Indeed, SCF deletion by genetic knockout in embryos led to lethality due to hematopoietic deficiencies. This pivotal study paved the way for studying the functional specificity of cells that make up HSC niches.

## 3. HSCs and Hypoxia

HSCs that reside in the vascular niche are short term, actively cycling and replenishing circulating cells by differentiating into hematopoietic cell types [38]. This metabolic state is thought to be due to the sinusoidal HSC niche being oxygen rich relative to the hypoxic endosteal niche (depicted in Figure 1), in which HSCs are mainly quiescent [39–41]. Hypoxia is necessary for the long-term maintenance of HSCs and is regulated by hypoxia-inducible factor (HIF)-1*α* [42]. The metabolic activity of these long-term HSCs is dependent on glycolysis, which is driven by Meis1 via transcriptional activation of HIF-1*α* [43]. The same HIF-1*α* stabilizes endosteal HSCs and maintains them in a state of quiescence, enabling them to withstand stressful conditions. This maintenance and survival of HSCs occurs via HIF regulation of vascular endothelial growth factor alpha (VEGF-*α*), Cripto/GRP78 signaling, and upregulation of CXCR4 [44, 45].

Osteoblastic cells, whose major role in the endosteal HSC niche has already been described, have been shown to regulate hematopoiesis by expanding the HSC pool and erythroid cells via a heretofore unknown ability to produce erythropoietin, which was previously thought to be produced only in the kidney [46]. This role is mediated by upstream HIF signaling in osteoprogenitors. Thus, HIF-1*α* is critical for the survival of HSCs.

## 4. Mesenchymal Stromal Cells

Mesenchymal stromal cells (MSCs) have the ability to differentiate in culture into multilineage precursors of bone, fat, and cartilage [47]. They provide a sustainable framework or scaffold in which the endosteal and sinusoidal HSC niches take root, as shown in Figure 1. Nestin expression identified a subset of MSCs that has been shown to be important in HSC niche formation [48]. These nestin-positive MSCs associate with HSCs and sympathetic nerve fibers. They express genes that maintain HSCs, and their depletion caused a proportionate drop in the HSC pool, suggesting an essential role in HSC niche formation. The interaction between MSCs and HSCs is mediated by N-cadherin [49]. MSCs have been shown to express agrin, a proteoglycan that plays a role at the neuromuscular junction, to enable hematopoietic cell proliferation [50]. Furthermore, MSCs have been shown to express CXCL12/SDF-1 ligand, which is crucial for HSC homing via CXCR4 [51]. This finding has been buttressed by the finding that HSCs are mobilized into the circulation by administration of CXCR4 antagonists [52]. MSCs are also able to form osteoblasts for the endosteum.

Despite the ability of MSCs to differentiate into multilineage cells *in vitro*, recent evidence suggests that the fate of MSCs may be restricted in bone marrow *in vivo*, with an ability to replenish only the osteogenic lineage, such as the osteoblasts that form part of the endosteal niche [53]. Therefore, the survival and maintenance of HSCs is tightly controlled by MSCs that associate with the HSC niche.

## 5. Chronic Myeloid Leukemia and LSCs

Chronic myeloid leukemia (CML) arises consequently to the reciprocal translocation of chromosomes 9 and 22, t(9:22), leading to expression of the fusion gene *BCR-ABL*. This gene encodes an oncoprotein that expresses a constitutively active tyrosine kinase and generates clonal leukemic cells [54, 55]. CML progresses through three major phases, from chronic phase to accelerated phase to blast crisis [56]. The chronic phase is the most treatment-responsive phase, while the blast crisis is an acute transformation of the disease process in which mature CML cells revert to immature forms that are insensitive to therapy, and can lead rapidly to host death. Identification of the *BCR-ABL* gene has led to specific targeting with tyrosine kinase inhibitor imatinib (Gleevec), which has achieved great success in depleting *BCR-ABL*-positive leukemia cells [57].

Actively cycling leukemia cells are especially vulnerable to imatinib therapy [58]. In contrast, quiescent leukemia cells are resistant to imatinib therapy [59, 60]. They persist in the bone marrow, constituting CML “minimal residual disease” and accounting for relapse or transition to the accelerated or blast crisis phase [61]. Newer tyrosine kinase inhibitors such as nilotinib or dasatinib are effective in eliminating *BCR-ABL* leukemia cells that have acquired additional mutations and keeping them in check. Like imatinib, however, these newer agents are unable to eradicate the quiescent CML cells [62]. Therefore, patients may have to be on chemotherapy for the rest of their life to prevent relapse [63]. Such long-term treatment poses major challenges, including uncomfortable side effects, costs, and noncompliance [64]. Thus, newer strategies that target not only the intrinsic regulatory mechanisms of residual leukemia cells, but also supporting factors that enhance their survival will be necessary for improved therapeutic efficacy and complete eradication of residual CML cells.

Understanding how CML minimal residual disease evades chemotherapy is best discussed in the context of CML stem cells. The CML cells that resist therapy have been shown to exhibit stem cell characteristics and are referred to as leukemia stem cells. LSCs are akin to HSCs in several ways. They can self-renew via wnt/*β*-catenin and hedgehog signaling, differentiate into different myeloid lineages that account for CML bone marrow pathology, and resist stressful conditions that threaten their survival [65–68].

A major hindrance in studying LSC biology is the lack of a clear method of detecting them in an unaltered bone marrow milieu. However, advancement in the knowledge of LSC biology has been made possible by harvesting leukemia cells from humans or mice, identifying the fraction of LSCs that meet the requirements for stem cell properties and using that fraction for *in vitro* and transplantation *in vivo* studies, whose purposes are to better understand the behavior of these cells and to apply them in developing new treatment strategies.

## 6. LSC Bone Marrow Niches

Like normal HSCs, LSCs are thought to harbor specialized microenvironments in the bone marrow cavity, including the endosteal and sinusoidal niches [69, 70] (illustrated in Figure 1). The endosteal niche has received more emphasis in LSC studies because the treatment-resistant cells are thought to lodge here and utilize metabolic programs that sustain their survival. Indeed, human or mouse LSCs have been transplanted *in vivo* and been shown to home to the epiphyseal osteoblastic surface of the endosteum before later dispersion to perivascular niches in vessels near the endosteum and diaphysis [71].

LSCs localize and associate with cells around the endosteum to form discrete niches. Other cell types, including MSCs, that contribute to this niche play a major role in LSC biology. In a pivotal study, Raaijmakers et al. showed that a specific deletion of Dicer1 in mouse osteoprogenitors prevents expression of the *SBDS* gene, a genetic mishap responsible for Scwachman-Bodian-Diamond syndrome, and gives rise to myelodysplasia and AML [72]. This suggests that genetic changes in bone marrow stromal cells that contribute to the endosteal niche are not trivial, because they are able to differentiate into osteolineage progenitors (osteoblast precursors) and initiate a malignant process in the normal HSC endosteal niche.

Importantly, this LSC endosteal microenvironment has been suggested to mirror the HSC hypoxic endosteal niche. In this context, BCR-ABL has been shown to induce, upregulate, and stabilize HIF-1*α* in CML stem cells [73, 74]. This enables the cells to survive in a quiescent state by undergoing metabolic changes, such as abandoning mitochondrial oxidative phosphorylation and switching to glycolysis [75]. We demonstrated that, unlike the HSCs, LSCs expand hypoxic bone marrow areas (depicted in Figure 1) and become resistant to chemotherapy. However, a hypoxia-activated dinitrobenzamide mustard, PR-104, reduced leukemic cell numbers and significantly extended host survival [76]. Thus, targeting hypoxia niches presents a novel opportunity for killing LSCs.

Using *in vivo* dynamic imaging, Sipkins et al. revealed that LSCs home and form perivascular niches in cranial bone marrow vasculature [70]. HSCs have been shown to use chemokine-mediated mechanisms such as CXCR4/SDF-1 to interact with the vasculature [51]. In fact, the interaction of LSCs with the vasculature was shown by Sipkins et al. to be strongly dependent on their expression of CXCR4 and binding to SDF-1 expressed by vessel endothelium. E-selectin was found to contribute to LSC vessel homing, but to a lesser extent than CXCR4. Interestingly, this site of LSC localization overlapped with the HSPC site. We demonstrated that, besides decimating CML cells, imatinib also upregulates CXCR4 in CML cells, helping them to migrate to shelter sites in bone marrow stroma, where they revert to a G_0_-G_1_ cell cycle state and survive therapy [77].

This upregulation of CXCR4 by imatinib was mechanistically dissected by showing a redistribution of CXCR4 in the lipid raft fraction of CML cells, where it colocalized with phosphorylated Lyn, suggesting that therapeutic targeting of the CML cell lipid raft is a viable option in preventing chemoresistance [78]. In another study, plerixafor, a CXCR4 antagonist, disengaged CML cells from bone marrow stroma and extracellular matrix and made them vulnerable to nilotinib therapy [79]. Yamamoto-Sugitani and colleagues showed that bone marrow stroma is capable of upregulating galectin-3, which caused activation of Akt and Erk, allowed accumulation of Mcl-1, and provided resistance to BCR-ABL tyrosine kinase inhibitors by subverting apoptotic induction [80]. Therefore, strategies that target these adhesion molecules may provide an opening for effective therapeutic tyrosine kinase inhibition.

Colmone et al. showed that LSCs adopted hematopoietic progenitor cell niches as “foster homes” and altered the residential dynamics of hematopoietic progenitor cells in a normal bone marrow microenvironment, as shown in Figure 1 [81]. The LSCs expressed stem cell factor, which enabled creation of new microenvironments, termed “malignant niches.” The new niches appeared to provide alternative homes for hematopoietic progenitor cells, distorting their migration patterns and dislodging them into the circulation on introduction of granulocyte colony-stimulating factor. Whether these new hematopoietic progenitor cell niches provide cues that may drive these progenitors toward malignancy is yet to be explored. In a recent study, Zhang et al. demonstrated that CML cells produce granulocyte colony-stimulating factor, which reduced expression of CXCL12 in CML bone marrow [82]. This made long-term HSCs present in CML bone marrow exhibit more mobility and reduced growth. However, imatinib reversed this effect and restored long-term HSC growth. Thus, LSCs appear to crosstalk with bone marrow stroma to secure their survival while preventing and outcompeting normal HSCs from benefiting from bone marrow resources.

In support of this crosstalk mechanism, a recent study showed that bone marrow stroma expresses high levels of placental growth factor in CML [83]. Previous studies have shown that BCR-ABL upregulates VEGF and induces angiogenesis to promote its survival [73]. However, Schmidt et al. demonstrated that stroma-derived placental growth factor, which also induces angiogenesis and enhances CML proliferation and metabolism, is independent of BCR-ABL regulation. Inhibition of placental growth factor was effective in prolonging survival of imatinib-sensitive and -resistant CML mice [83], thus identifying another target that is crucial in the survival and growth of CML.

## 7. Conclusions

We have highlighted the successes and obstacles in combating LSCs, drawing from parallels in HSC studies. Several new targets have been identified within the supporting bone marrow microenvironment; the challenge lies in finding therapies that can specifically address these targets in a combinatorial manner with other therapies targeting intrinsic pathways. More needs to be accomplished to develop novel therapeutic strategies that can completely eradicate residual LSCs. Recent discoveries indicate that elucidation of the molecular mechanisms of leukemia-microenvironment interactions will provide a framework for the identification of novel-targeted therapies aimed at destroying LSC without adversely affecting normal stem cell properties. Finally, it may be worthwhile to determine the role of the immune system in LSC biology, especially as immunotherapy of solid tumors is gaining prominence.
